# Covid-19: food insecurity, digital exclusion and Catholic schools

**DOI:** 10.1007/s40839-020-00112-8

**Published:** 2020-09-19

**Authors:** Stephen J. McKinney

**Affiliations:** School of Education, University of Glasgow, Glasgow, UK

**Keywords:** Covid-19, Catholic schools, United Kingdom, Food insecurity, Digital divide

## Abstract

Covid-19 and the subsequent worldwide lockdowns have had a major impact on families and school education. The lockdowns have highlighted and exacerbated the disadvantages experienced by those children who suffer from child poverty. This article focuses on food insecurity and the digital divide, or digital exclusion, and argues that these have emerged as very pressing issues during lockdowns for children suffering from child poverty. The article provides an outline of the response of the Catholic Church and Catholic schools, primarily in the United Kingdom. There have been some concerted efforts to address food insecurity by providing food and food vouchers for children and vulnerable families. It has proved more problematic to address digital exclusion and the article argues that for those children who experience digital exclusion, this can effectively mean exclusion from the religious education, religious life, community and the pastoral and spiritual support that is normally offered by the Catholic school.

## Introduction

Catholic schools throughout the world, like other schools, have faced unprecedented challenges caused by the Covid-19 lockdowns. There has been a widespread closure of school buildings and a move to home education and online learning and teaching. In some cases, this has created serious financial difficulties and some Catholic schools in America, for example, which were partly dependent on income from parishes, were closed permanently during lockdown and others will not reopen after the lockdown (Reis 2020; Sparks 2020). When schools were temporarily closed down as a result of the lockdowns, there was a highly publicised move to home schooling, or more accurately, home education. The move to home education was an unexpected situation and not a choice. The home-schooling movement, by contrast, is composed of parents and carers who have chosen to educate their children at home and have planned and prepared for this educational process. They have chosen this option for various reasons including dissatisfaction with the school environment, the provision of religious education and the standards of academic engagement (Jolly and Matthews 2020). Parents and carers under lockdown had not chosen home education and had little time to plan and prepare. Many found home education challenging, felt ill equipped in terms of knowledge and skills and struggled with the change in relationship with their children (Meredith 2020; The Congregation for Catholic Education 2020). Many parents and carers had to balance the time for educating their children with the time required to work at home (BBC News 2020).

While the effects of lockdown have disrupted the education of many children it has also had a serious effect on the health and wellbeing of children as they have attempted to negotiate restrictions on their movement and the absence of physical contact with their friends. As noted above, there has been a confusing and difficult recalibration of familial relationships as parents and carers have attempted to adopt additional roles as home educators. An increasing number of households and children were suffering from the effects of limited household income before Covid-19 and the lockdowns in different parts of the United Kingdom. The effect of the lockdown has been especially detrimental to children who are living in poverty. The figures for child poverty in the United Kingdom are that 4.2 million children (around 30% of all children) were recorded as living in poverty in 2018–2019 (Child Poverty Action Group 2020). It is important to note that there are some small variances in this universal figure across the four constituencies of the UK and there are differences in the way the four constituencies address child poverty and the effects on education (Thompson and Ivinson 2020). Children are dependents and their poverty is normally related to the poverty of their household (McKinney 2014). This is usually determined by limited household income which means that decisions have to be made about how the income is used and which essentials such as food, heating, shelter and clothing need to be prioritised over others. Many households on limited income depend upon means tested free school meals to help feed their children, and there was a sharp rise in households accessing foodbanks before Covid-19. There may not be enough income for the family to support the technology and internet access to engage with the digital world.

This article will examine the two issues of food insecurity and the digital divide in the period of lockdown caused by Covid-19, how these have affected children from backgrounds of poverty and disadvantage and the response of the Catholic Church and Catholic schools. These two issues have been selected because food insecurity was becoming a serious issue pre-lockdown and the lockdown has created greater awareness of the existence of digital exclusion and the educational and social consequences of digital exclusion for many children and young people. The article will begin with a concise overview of Catholic Social Teaching, Catholic schools and poverty. The article will then explain that food insecurity and the digital divide are very serious manifestations of household poverty and that the lockdown has exacerbated these two issues. The article will outline some of the responses by the Catholic Church and Catholic schools to support children suffering from these disadvantages. The scope will include examples from different parts of the world but with a principal focus on the United Kingdom. The article will argue that the lockdown has had a serious effect on the religious education, religious life, community and the pastoral and spiritual support that is normally offered to practising, intermittent and lapsed Catholic children (and non-Catholic children) by the Catholic school. Where there has been some online support provided this has unfortunately excluded those children who do not have access to online resources, unless materials were downloaded for them.

## Catholic social teaching, Catholic schools and poverty

Catholic Social Teaching is unequivocal in responding to the social evil of poverty. Jesus established the Kingdom of God that would enable a new social order of justice to the poor, release of the oppressed, and the consoling of the afflicted (Pontifical Council for Justice and Peace 2005, section 325). This is expressed in the twentieth and twenty-first centuries as the preferential option for the poor. The preferential option is rooted in the gospels, especially the gospel of Luke, and is highly prominent in contemporary Catholic Social teaching. The teaching of the recent popes reflects this prominence (McKinney 2018). Gustavo Gutiérrez has argued that Christians cannot separate their discourse on God from the everyday experience of the life of the poor (Gutiérrez 1999). Christians cannot accept a broken world of social injustice which is a dark shadow of the good world that God created.

How do Catholic schools respond to poverty, child poverty, food insecurity and the digital divide? Some caution must be exercised here as ‘Catholic schools’ is a generic term that does not refer to a homogeneous group of ‘Christian ethos’ schools (Francis et al. 2018). There are variations across and within national cultural contexts that pertain to teacher profile, student profile, funding model and position within state conceptions of school education. This applies to the Catholic schools within the UK as much as it applies to comparisons between the UK and other parts of the world. These variations notwithstanding, there are three levels of appropriate response: theological, educational and social action. Historically, many contemporary Catholic schools were established in the UK in the nineteenth century with a mission to educate the poor (Grace 2002, 2003; McKinney 2019). In the Conciliar and post-Conciliar era, a number of the key Vatican documents on Catholic education have emphasised the role of Catholic schools in caring for the poor which, reflecting the position of the contemporary Church, became articulated as the preferential option of the Catholic school for the poor (Pope Paul VI 1965; The Sacred Congregation for Catholic Education 1977; Congregation for Catholic Education 2002). This theological response of the preferential option for the poor is the foundation for the educational and the social response.

Education is, of course, an important part of the response to ensure that young people understand these socially unjust situations and the causes and effects of these situations (Grace 2013; McKinney 2020). In normal circumstances, and even online under lockdown, Catholic schools can highlight the social injustices of poverty and hunger as part of the curriculum in the classroom (whether physical or virtual). The pupils can study the sociological and economic causes of child poverty and hunger and the crippling effects of hunger and engage in theological and ethical reflection drawing on contemporary Catholic Social Teaching (Byron 2015). This study can also include examination of the digital divide and the effects of the digital divide on the learning of some young people and their preparedness for future study and employment. This study and engagement will aim to develop a theologically literate group of young people able to articulate a contemporary Christian response to these pressing issues and actively seek transformative solutions.

There was also an immediate and urgent social response required to address the fact that many children, before the lockdown, were arriving to the school in the morning and were hungry. They had nothing to eat or not enough to eat to sustain them through the morning. Many Catholic schools had responded to this by feeding these children; the practical work of feeding the hungry that is called for in Matthew 25:31–46 (Gutiérrez 1983). Similarly, some young people were unable to engage with online learning and teaching at home because the family lacked the necessary equipment or access to the internet. Catholic schools, like many other schools, attempted to support these children with extra access to the internet at school. The effects of food insecurity and the digital divide for some children became worse under the lockdown.

## Food insecurity

The second Sustainable Development Goal of the United Nations is to achieve ‘zero hunger’, to bring about the end of hunger by 2030 (FAO 2020). Food security is accomplished when people have enough food that is nutritious and enables them to lead a healthy life (Global Food Security 2020). The contemporary challenge is to eliminate the experience of hunger and undernutrition for many but also the dangers of overweight and obesity that is damaging the health of others. Food insecurity means that people do not have access to adequate food that is nutritious, or they have sporadic or limited access to food in terms of quantity and quality (Taylor and Loopstra 2016). The food in the world is unevenly distributed and there needs to be strategic planning to ensure sustainable food production that will meet the requirements of food security. The contemporary Catholic Church has repeatedly condemned the unjust global distribution of food. The Church understands food insecurity as a serious impediment to the human right to life and human dignity as it is a denial of the means to support that life and dignity—a dignity that is God-given (United States Conference of Catholic Bishops 2020). One of the most recent statements is from Pope Francis (2019) who argues:

It is a cruel, unjust and paradoxical reality that, today, there is food for everyone and yet not everyone has access to it, and that in some areas of the world food is wasted, discarded and consumed in excess, or designed for other purposes than nutrition.

Food insecurity for children in school is often partially addressed by the provision of free school meals for those children who belong to families on low incomes. This is a threshold benefit and is used throughout the United Kingdom. An increasing number of families on low income have also used foodbanks. The uptake at foodbanks pre-Covid-19 had increased dramatically as people struggled with lower household incomes often as a result of Universal Credit. The Universal Credit benefit was introduced in 2010 and was intended to streamline benefits but has caused major problems for vulnerable families because of the initial delay in the first payment and the means of assessing subsequent payments that can result in fluctuations in the amount of the monthly payment (Miller and Bennett 2017). Foodbanks were allowed to remain open throughout the lockdown.

The history of contemporary foodbanks has strong roots in Christian activism and one of the first contemporary foodbanks can be traced to 1967 in Phoenix, Arizona in the United States (Riches 2002). This was set up by John van Hengel, a devout Catholic. His colleague Robert McCarty set up foodbanks in Canada and foodbanks began to spread throughout Europe in the 1990s (Pemberton 2020). A number of Christian denominations became involved in establishing foodbanks and the Trussell Trust (one of the largest foodbanks networks in the UK alongside Fareshare) was founded in the UK by two Christians, Carol and Paddy Henderson in 1997. The Trust initially sought to help homeless children in Bulgaria but began to work to reduce food insecurity in the UK when they opened the Salisbury foodbank in 2000 (Trussell Trust 2020a). The recession caused by the economic meltdown in 2008 prompted a marked increase in use of foodbanks (Riches and Silvasti 2014). There has been another marked increase during the period of lockdown. The Trussell Trust reported an 89% increase in the uptake of emergency food parcels in April 2020 compared to April 2019 (Trussell Trust 2020b). This included a 107% rise in parcels for children and the number of families with children seeking food parcels has almost doubled compared to April 2019. The Independent Food Aid Network reported a 175% increase in April 2020 compared to April 2019. The salient point here is that foodbanks preceded the recession of 2008 and the outbreak of Covid-19 and the lockdown, operated throughout the lockdown, and will continue once the lockdown has been discontinued.

The UK government introduced some emergency measures in England to support vulnerable families that depend on free school meals during the lockdown. These measures consisted of the provision of meals, food parcels and food vouchers (Department of Education 2020). There were serious difficulties for some families when they attempted to redeem food vouchers (Burns 2020). Other families found themselves vulnerable because the adults had lost their jobs or struggled to survive on the 80% reduction in the furlough scheme (Caritas Westminster 2020). The continued lockdown caused by Covid-19 has resulted in a greater number of families that have become vulnerable and an increasing number of children who are experiencing child poverty. The food vouchers have been extended for eligible children into the summer holidays (Lawrie 2020). The UK government recognises that more children may become eligible over the summer period as a result of their families becoming vulnerable (GOV.UK 2020a).

Before the outbreak of Covid-19, the Catholic Church responded in different parts of Britain to food insecurity and supported children in Catholic schools. In the Archdiocese of Westminster 24 parishes were directly supporting a foodbank and another 56 were helping foodbanks in 2019 (Gledhill 2019). This is about one third of the parishes in the Archdiocese.

The Brentwood Catholic Children’s Society, established to deliver mental health and emotional wellbeing services in Essex and East London, issued around 100 food vouchers per year before the outbreak of Covid-19. The Society supports all children regardless of background, race or religion (Brentwood Catholic Children’s Society 2020). The effects of the lockdown have resulted in the most vulnerable families becoming more vulnerable and in June 2020, Caritas Westminster reported a significant increase in demand for food from foodbanks and food relief projects (Teague 2020). Caritas Westminster has responded to this by providing its own food voucher scheme to support vulnerable families and those families that have been unable to redeem the government vouchers (Caritas Westminster 2020). In the first phase they supported 62 parishes and schools, and this was extended in the second phase to 72 parishes and schools including 6 parishes in the Diocese of Southwark. Brentwood Catholic Children’s Society has noted a rise in demand for food vouchers. Catholic Care, based in the Diocese of Leeds, started to provide fresh fruit in June 2020 to vulnerable children who were still attending schools in the lockdown (Catholic Care 2020).

In Scotland there are no separate figures for free school meals for Catholic schools readily available, but the state-funded Catholic schools educate approximately 120,000 of the children in Scotland. Many of the Catholic schools are concentrated in the post-industrial areas in Scotland that record high levels of poverty and deprivation according to the Scottish Index of Multiple Deprivation (Scottish Government 2020a). This includes the City of Glasgow, West Dunbartonshire, Inverclyde and North Lanarkshire. The Scottish Government introduced free school meals for all children from primary one to primary three in January 2025 (Scottish Government 2020b). After primary three free school meals entitlement is a means tested benefit (McKendrick et. al. 2019). The number of children receiving free school meals prior to the lockdown was 122,000 (Scottish Government 2020c). The Scottish Government has reported that the figure of 122,000 children receiving free school meals has increased to 175,000 and has pledged further funding to extend the provision of free school meals into the summer holidays (my.gov.scot 2020). The St. Nicholas Care Fund was established in the Archdiocese of Glasgow in 1992 to provide help for the poor in the Glasgow area. The Fund had awarded £35,000 to schools, churches and community organisations by mid-May 2020, in response to the hardship caused by the lockdown. Most of the money was used to help feed families and some of the funding was used by Catholic secondary and primary schools to provide food for families (Swanson 2020).

## The digital divide

The digital divide or digital exclusion is emerging as another manifestation of poverty that has been exacerbated by the effects of Covid-19. Technology is used to access information, to acquire services and has had a big impact on communication and working practices (Office for National Statistics 2019). In the UK, the essential digital skills framework, an update on the Basic Digital Skills framework of 2015, was published in 2018 (Lloyds Bank 2018). The essential digital skills for life and work for adults in the UK is measured using the framework of five skills categories. The five skills categories are: managing information; communicating; transacting; problem solving and being safe and legal online (Lloyds Bank 2020). Managing information means searching for information online (GOV.UK 2018). Communicating includes messaging using an online service. Transacting refers to purchasing items or services online. Problem solving is verifying online information and solving problems with digital devises/apps. Being safe online means that an adult is aware of the risks and threats of using the internet and is aware of the need for security measures. An adult is considered to have full digital skills if she/he could perform a task in each of the skills categories. There is a marked low digital engagement in the older age groups in the UK. In the over 70 age bracket, 77% have low digital engagement. The figure for the 60–70 age bracket is 52%. Those who are most disadvantaged in society are more likely to have low digital engagement. The overall figure for the population lacking digital skills is 22%. The figure for households on low income of £6000–£10,000 having internet access is 51% compared to 99% of households of an income of over £40,001 (Holmes and Burgess 2020).

The use of technology in schools in the UK is increasing rapidly to reflect changes in society and pupils are now expected to acquire the new education and life skill of digital literacy (Phelan 2020). The access to equipment and development of skills are high priorities. A pilot study in Scotland provided teachers and pupils with a personal iPad and this increased pupil motivation, interest and engagement with technology and promoted greater pupil autonomy and greater responsibility for their own learning (Burden et al. 2012). The outbreak of Covid-19 that led to lockdown has highlighted and deepened existing inequalities such as digital exclusion for some school children. There can be a digital divide because of the technological expertise and resources available in a particular school compared to another school and there can be a digital divide in the technological resources available in the home. There were issues about the readiness of some teachers with low digital engagement, who did not have developed digital skills and were required to teach and engage online. Further, some schools did not have all of the resources required for online learning and teaching before Covid-19 (Roach 2020).

There are serious concerns about the education of the most disadvantaged children who have been unable to engage or fully engage with school education online because of digital exclusion. These children have not had access to appropriate devices or have had to share devices with other members of the family (Holmes and Burgess 2020). There may have been problems with connectivity and the quality of the Broadband connection. The parents may not have had the necessary skills to support the online learning in the home. There have been times when some families could not afford to pay the wi-fi bill as the limited household income needed to be used for items such as food or heating. There were steps taken to try to address digital exclusion. There was an announcement by Gavin Williamson, the Education Secretary, in April 2020, that devices such as laptops and tablets, and 4G routers would be provided for disadvantaged children In England (GOV.UK 2020b). This provision was intended for those in the most vital stages of their education. This scheme suffered delays and there was confusion over accessing the support. Later in June a bill was introduced in Parliament that proposed that all children eligible for free school meals (1.3 million) should be provided with a broadband connection and devices (Halliday 2020).

In Scotland, the initial plans for a phased return to school and blended learning was to take into account the digital access that children have and this was to be part of the decision making about who returned to school earlier and who remained in school for a longer period of time in the week (Scottish Government 2020d). In later announcements there were plans to commit £30 million to providing laptops to disadvantaged children and there was an awareness of the connectivity issues (Swinney 2020).

The effects of digital exclusion affected many disadvantaged children who attend Catholic schools in different parts of the world. In New South Wales, Australia, the Chief Executive of Catholic schools expressed concern about the geographical and social digital divide in out-of-school and online learning (Cath News 2020). The Western Region Catholic Foundation for Education in India oversees Catholic schools that are ‘budget’ or government aided. The Foundation helped teachers to prepare to teach online and established a strategy to support the children. Many of the parents did not have a spare device to share with the children in the period of lockdown and so the Foundation set up a resource bank where people could donate a device that is not required (Borwankar 2020).

## The effects of digital exclusion on religious engagement and the Catholic school

While the Catholic Church warns of the dangers and risks of the internet and the use of social media, including the digital divide which excludes the poor, the internet and social media are understood to be important modes of contemporary communication (Pontifical Council for Social Communications 2002a, b). The title of an article by Cairns and Gledhill in the Tablet dated the 18th of June is *Isolated but not alone: resources for Catholics* (Cairns and Gledhill 2020). This is an inventory of the impressive response to Covid-19 by the Catholic Churches and Catholic organisations across the UK. The ‘resources’ include numerous opportunities to participate in on-line masses and prayer, listen to sacred music, hear sermons and talks. In Scotland a number of attractive and engaging on-line resources for religious education for Catholic schools were produced in response to Covid-19 (Scottish Catholic Education Service 2020). These could be used in a classroom setting, in the home or as part of blended learning. Some spiritual care resources were produced as the children returned to school in early August 2002.

The most disadvantaged Catholic families that are digitally excluded would not have been able to access these resources online. This means that they would have been unable to participate in Sunday mass and would have missed the Easter Triduum in its entirety. This raises serious questions about the opportunities for the children in these families to access religious education, religious life and the community offered by the Catholic school. The children would not have been able to access any online education, religious education resources (unless downloaded for them by the school) and engage in any formal religious practice.

There are, of course, different ways to categorise the pupil population of Catholic schools (Mclaughlin et al. 1996; Village and Francis 2016). This article will adopt the following: practising, intermittent, lapsed and non-Catholic and adapt the explanations of the categories proposed by Village and Francis (2016). Practising Catholics attend church every Sunday, intermittent Catholics attend church but not every Sunday and lapsed Catholics never attend Church on a Sunday. The children who are practising normally engage in weekly religious practise in a local Catholic parish and also engage in religious practise through the Catholic school. The children who are intermittent Catholics engage in weekly mass in an occasional way and probably engage in religious practise more regularly through the Catholic school. The lapsed Catholics never attend weekly mass but do have opportunities to experience Catholic religious practise in the Catholic school.

The lockdown demonstrates how important this engagement through the Catholic school has been, and will be, once the Catholic schools re-open. The children who practise and experience digital exclusion will not have participated in any online prayers and masses nor in any religious education through the school. It is reasonable to assume that some of these families may have introduced or enhanced forms of home prayer or devotion. If the Catholic school is the only engagement, or the main source of engagement, the intermittent and lapsed children have with religious life and the community of the Catholic school, there will have been no engagement through the period of lockdown.

The children who have limited or no access to the internet may also have had no opportunity to access, or had limited access to, the pastoral and spiritual support that is offered by the Catholic school. This pastoral support is especially important for those children of families which are fragile or have become broken (Congregation for Catholic Education (1997, p. 5). An increasing number of families have become fragile since the lockdown as adults have lost jobs or have struggled with reduced incomes. There are anxieties about all children who live in situations of neglect and those who suffer emotional, physical or sexual abuse in the home (Wodon 2020; Belcher 2020). In many cases the children have been isolated with the abusers (Javid 2020). There are very concrete plans being laid for the pastoral support of children returning to school across the UK and there will need to be a very careful focus on the pastoral support in Catholic schools (Gov.UK 2020c).This will be required for children who had a pre-Covid-19 history of pastoral needs and for children attempting to cope with the effects of the loss of employment and financial security in the home during the lockdown. There are many children who will have suffered stress, anxiety and depression in lockdown (NSPCC 2020).

## Concluding remarks

This article has highlighted the effects of the lockdown caused by Covid-19 on school children and has focussed on the food insecurity and the digital divide or digital exclusion on Catholic schools. This article presents a snapshot, a moment in time, but it is also a moment in a deeply troubling cycle of the effects of poverty and deprivation. The adverse effects of food insecurity and the digital divide existed before Covid-19, have become exacerbated under the lockdown, and will continue to be harmful to the education of children and their long-term future after the lockdown. There are anxieties about the future for all young people. There is concern that a deep recession will be triggered by the lockdown. In the UK, young people under the age of 25 are more likely to have lost their jobs during the lockdown. Many of them were working in low paid jobs in accommodation, food and retail and may find it difficult to gain new employment after the lockdown (Dias et al. 2020). Some universities in the UK face severe cuts in income, potential job losses and possibly insolvency (Drayton and Waltman 2020). This is alarming news as universities which generate skills and upskill and reskill members of the workforce, are key to recovery for the economy and for society (Scottish Government 2020e).

A number of Catholic schools have attempted to respond to the challenges of lockdown, to the challenges of food insecurity and the digital divide as best they can. They have responded with social action that is rooted in the theological preferential option for the poor. They have drawn on the resources that are available to them including support from the Catholic Church. Much has been learned that will prove invaluable in the event of subsequent lockdowns in national or regional areas. This will be especially the case in the countering of further effects of the digital divide. The Catholic schools will now face a further set of challenges as they prepare young people for a positive and viable school leaver destination in the post-lockdown reality.
